# Topology-Optimized Kirigami Design of Electrospun BNNS/PVA Composite Films for Flexible Electronics Thermal Management

**DOI:** 10.3390/nano16150926

**Published:** 2026-07-28

**Authors:** Yanyan Xu, Bin Xie, Mingxiang Chen, Xin Tang

**Affiliations:** 1 China-EU Institute for Clean and Renewable Energy, Huazhong University of Science and Technology, Wuhan 430074, China; m202471777@hust.edu.cn; 2 School of Mechanical Science and Engineering, Huazhong University of Science and Technology, Wuhan 430074, China; m202470937@hust.edu.cn

**Keywords:** flexible electronics, thermal management, BNNS/PVA composite film, topology optimization, electrospinning, kirigami

## Abstract

Flexible electronics require thermal-management materials that can efficiently dissipate heat while maintaining mechanical compliance under deformation. In this study, a topology-optimized kirigami BNNS/PVA composite film was developed by combining boron nitride nanosheet incorporation, electrospinning, and thermo-mechanical topology optimization. The electrospun BNNS/PVA network enhanced the in-plane thermal conductivity from 0.19 to 4.63 W/(m·K), while the optimized kirigami architecture improved deformation accommodation by reducing elastic strain energy accumulation. The average temperature and total elastic strain energy were reduced from 86 °C to 53 °C and from 43 J to 11 J, respectively. The optimized structure achieved a maximum stress of 2.39 MPa and a maximum strain of 5.02% and reduced the steady-state temperature by up to 21.27 °C under identical heating conditions. Furthermore, in-plane thermal conductivity was maintained after 300 bending cycles, which provides an effective material–structure design strategy for flexible electronic thermal management applications.

## 1. Introduction

The rapid development of flexible electronics [1], wearable devices [2,3], soft robotics, and deformable sensors has created increasing demand for composite films that can simultaneously provide efficient heat dissipation and mechanical deformability [4]. However, mechanically compliant polymer materials, such as polydimethylsiloxane (PDMS), polyurethane (PU), thermoplastic polyurethane (TPU) and poly(vinyl alcohol) (PVA), can provide flexibility [5,6,7,8,9], while most polymers have an intrinsic thermal conductivity of only 0.1–0.5 W/(m·K) [10], which severely restricts heat dissipation. As a consequence, insufficient thermal transport in flexible polymer composite films can induce local heat accumulation during repeated deformation. Previous studies have also shown that maintaining stable heat dissipation during bending–releasing cycles is essential for flexible thermal management [11]. Therefore, achieving high thermal conductivity without sacrificing structural stretchability remains a critical challenge for flexible thermally conductive composite films [12].

To improve the thermal conductivity of polymer composites, boron nitride nanosheets (BNNS) have been widely incorporated into polymer matrices as thermally conductive fillers [13]. BNNS are particularly attractive for flexible thermally conductive composite films because of their high intrinsic thermal conductivity (can reach up to approximately 2000 W/(m·K) [14]), excellent electrical insulation and chemical stability [15,16,17,18,19], which is favorable for constructing in-plane heat-conduction pathways. Nevertheless, many existing approaches still rely on high BNNS loading or dense thermal networks, which improve thermal transport at the expense of stretchability and mechanical compliance [20,21,22]. For instance, Zhao et al. [23] reported boron nitride nanosheet/aramid nanofiber (BNNS/ANF) films with high in-plane thermal conductivity, while the single-layer film at the same BNNS content showed a tensile strength of only 36.7 MPa. By constructing a multilayer gradient structure, the tensile strength increased to 59.3 MPa, and the in-plane thermal conductivity reached 19.13 W/(m·K). This result indicates that rigid filler networks require rational structural design to mitigate mechanical degradation.

In this work, an electrospun BNNS/PVA composite film integrated with a topology-optimized (TO) kirigami structure is developed to achieve a balance between in-plane thermal conductivity and stretchability. The incorporation of BNNS into the PVA matrix, together with the electrospun fibrous architecture, facilitates the formation of an in-plane heat-conduction pathway [24]. In parallel, the TO kirigami design improves tensile deformability by regulating material distribution and reducing elastic strain energy accumulation. This combined material–structure strategy enables efficient heat dissipation and enhanced stretchability in flexible polymer composite films.

## 2. Materials and Methods

Introducing 2D thermally conductive fillers is an effective strategy to enhance the in-plane thermal conductivity of flexible polymer composites [25]. In this work, BNNS were selected as the thermally conductive filler, while PVA was used as the flexible polymer matrix. BNNS were dispersed into the PVA aqueous solution to prepare a homogeneous spinning solution. Subsequently, electrospinning was employed to fabricate BNNS/PVA fibrous composite films, where the stretching of the charged jet and directional collection promoted the in-plane arrangement of fibers and facilitated the formation of continuous heat-conduction pathways. After drying, the composite film was further processed into a kirigami structure by laser cutting to improve stretchability.

### 2.1. Materials

#### 2.1.1. Boron Nitride Nanosheets

Boron nitride nanosheets (BNNS, particle size: 0.1–0.4 μm, 98 wt%) were purchased from 3AChem Co., Ltd., Shanghai China. BNNS were used as the thermally conductive filler to enhance the heat-conduction capability of the composite films.

#### 2.1.2. Poly(Vinyl Alcohol)

Poly(vinyl alcohol) (PVA, alcoholysis degree: 98.0–99.0%, LR grade) was supplied by Macklin Biochemical Co., Ltd., Shanghai, China. PVA was used as the polymer matrix for preparing the flexible BNNS/PVA composite films.

#### 2.1.3. Deionized Water

Deionized water was used as the solvent for preparing the PVA aqueous solution and BNNS/PVA spinning solution throughout this work.

#### 2.1.4. Graphene Heating Film

A commercially available graphene heating film, supplied by Kangre, Nanchang, Jiangxi Province, China, was used as a flexible heat source to evaluate the heat dissipation efficiency of the BNNS/PVA composite films.

#### 2.1.5. 2D Servo Motor System

A 2D servo motor system equipped with DS3218MG digital servos (Ruosi Technology Co., Ltd., Shenzhen, China)was used to simulate the repeated bending motion of a robotic arm joint. This setup provided a dynamic deformation condition for evaluating the thermal stability and deformation adaptability of the BNNS/PVA composite films under repeated bending.

### 2.2. Preparation of BNNS/PVA Spinning Solution

**Figure 1:**
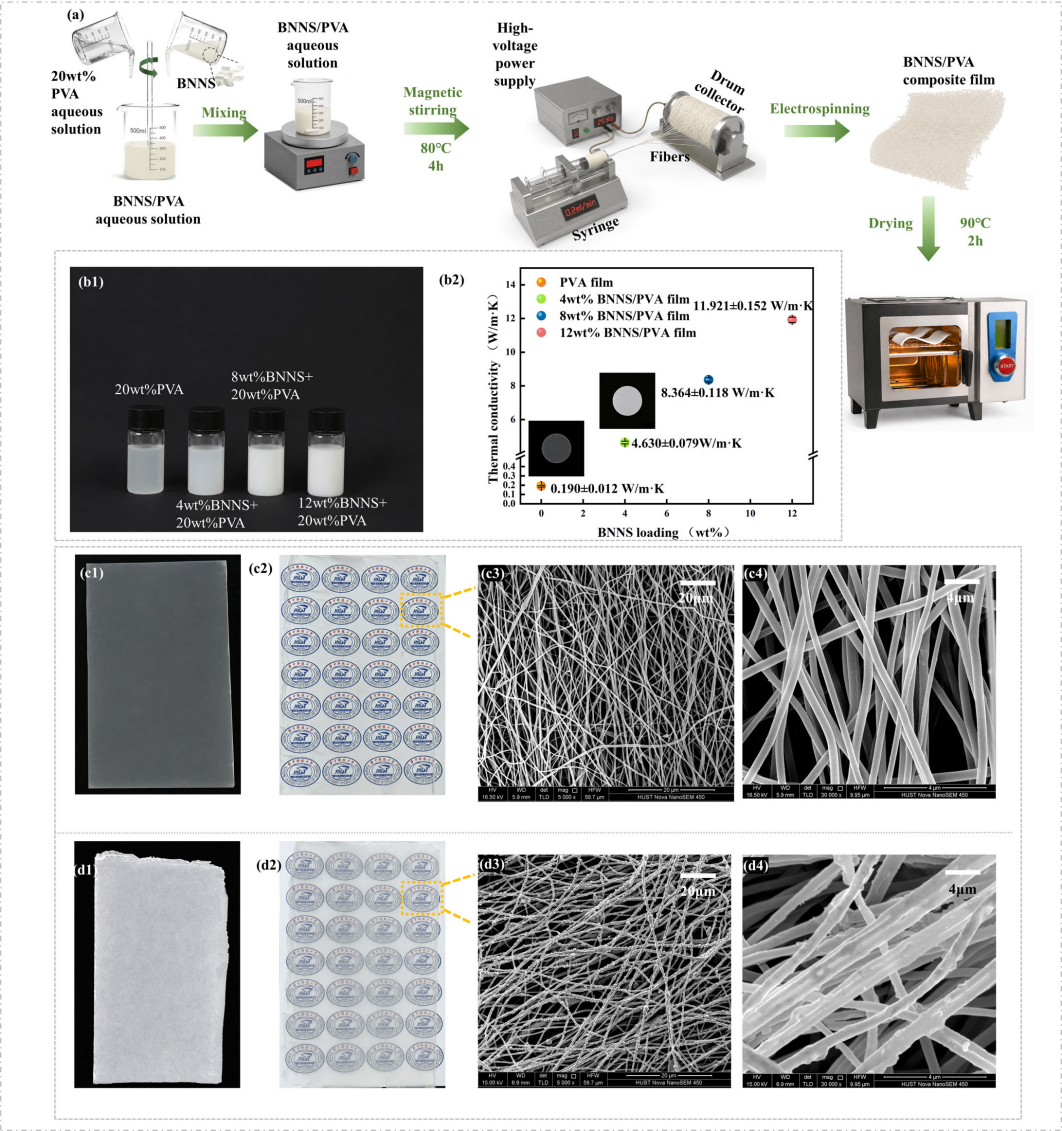
Fabrication and characterization of electrospun BNNS/PVA composite films. (**a**) The BNNS/PVA composite films were prepared through solution mixing, magnetic stirring, electrospinning, and drying. (**b1**) Optical images of PVA and BNNS/PVA spinning solutions with different BNNS loadings. (**b2**) In-plane thermal conductivity of the films with increasing BNNS loading. (**c1**,**c2**) Optical images of the electrospun PVA film. (**c3**,**c4**) SEM images of the PVA film. (**d1**,**d2**) Optical images of the 4 wt% BNNS/PVA composite film. (**d3**,**d4**) SEM images of the 4 wt% BNNS/PVA composite film.

The BNNS/PVA spinning solution was prepared by dispersing BNNS into a PVA aqueous solution. As shown in Figure 1a, a 20 wt% PVA aqueous solution was first prepared using deionized water as the solvent. Then, 4 wt% BNNS was added under continuous stirring, followed by magnetic stirring at 80 °C for 4 h to fully dissolve PVA and uniformly disperse BNNS. The obtained BNNS/PVA solution was used as the spinning solution for electrospinning.

### 2.3. Electrospinning of BNNS/PVA Composite Films

Electrospinning was selected to fabricate BNNS/PVA composite films because it can generate continuous polymer fibers under a strong electric field and extensional flow. During this process, the stretching of the charged jet and the directional collection on a rotating drum are beneficial for constructing an oriented fibrous architecture, which can promote the in-plane arrangement of BNNS and facilitate continuous heat-conduction pathways in the composite film.

The prepared BNNS/PVA spinning solution was transferred into a syringe and fed into the electrospinning system using a syringe pump. A high voltage of 26 kV was applied between the needle and the grounded rotating drum collector. Under the electric field, the droplet at the needle tip formed a Taylor cone and was stretched into a continuous charged jet. With solvent evaporation, BNNS/PVA fibers were formed and directionally deposited on the rotating drum collector.

After electrospinning, the BNNS/PVA composite film was dried at 90 °C to remove residual moisture and stabilize the film structure. The obtained electrospun fibrous network provided the structural basis for enhanced in-plane thermal transport in the BNNS/PVA composite film.

### 2.4. In-Plane Thermal Conductivity Characterization of BNNS/PVA Films

As shown in Figure 1(b1), the BNNS/PVA spinning solutions exhibited increasing opacity with increasing BNNS loading, indicating the gradual incorporation of BNNS into the PVA matrix. The 4 wt% BNNS/PVA solution remained relatively homogeneous without obvious sedimentation or macroscopic aggregation, suggesting good dispersion stability and processability for electrospinning. In contrast, higher BNNS loadings led to more concentrated and opaque suspensions, which may increase the viscosity of the spinning solution and induce instability during electrospinning.

The in-plane thermal diffusivity of the electrospun BNNS/PVA composite films was measured using a laser flash analyzer (LFA 467, NETZSCH, Selb, Germany) equipped with an in-plane outward-heat-flow sample holder. Laser flash analysis has been widely employed for evaluating thermal diffusivity in polymer-based materials and anisotropic composites due to its capability of characterizing transient heat transport behavior. The relationship between thermal diffusivity and thermal conductivity can be established through the equation [26]:

(1) $$k=\alpha \rho C_{p}$$

where *α* is the measured in-plane thermal diffusivity, *ρ* is the density of the specimen, and *C_p_* is its specific heat capacity.

Circular specimens with a diameter of approximately 25.2 mm and a thickness of approximately 0.124 mm were used for the measurements. The effective detection diameter was 14.0 mm. During the test, nitrogen was supplied as both the purge and protective gas at a flow rate of 50 mL min^−1^. The thermal response was detected using an MCT detector, and the data were analyzed using NETZSCH Proteus software (Version 7.0) with the in-plane isotropic model and pulse correction.

At each test temperature, three flash measurements were conducted, and the average thermal diffusivity and standard deviation were calculated. For example, at 70 °C, the electrospun film prepared from the 4 wt% BNNS/PVA solution exhibited in-plane thermal diffusivities of 4.698, 4.543, and 4.648 mm^2^ s^−1^, giving an average value of 4.630 ± 0.079 mm^2^ s^−1^.

As shown in Figure 1(b2), the in-plane thermal conductivity increased from 0.190 ± 0.012 W/(m·K) for the neat PVA film to 4.630 ± 0.079, 8.364 ± 0.118, and 11.921 ± 0.152 W/(m·K) for the 4 wt%, 8 wt%, and 12 wt% BNNS/PVA films, respectively. Although higher BNNS loadings further enhanced the MPA thermal conductivity, the 4 wt% BNNS/PVA film was selected for subsequent experiments because it provided a pronounced in-plane thermal conductivity enhancement while maintaining favorable dispersion stability, electrospinning processability, and film flexibility [27]. This balance is particularly important for the following kirigami-based structural design and flexible thermal management applications [28].

### 2.5. Characterizations

The morphology of the electrospun PVA film and 4 wt% BNNS/PVA composite film was characterized by optical images and SEM. As shown in Figure 1(c1,c2), the pure PVA film exhibits a smooth, uniform, and highly transparent appearance, indicating good film-forming ability after electrospinning. The SEM image at a scale of 20 μm in Figure 1(c3) shows that the PVA fibers are randomly interwoven to form a continuous fibrous network. At a higher magnification with a scale of 4 μm in Figure 1(c4), each PVA fiber displays a smooth and continuous surface, without obvious particles, beads, or structural defects.

After the incorporation of 4 wt% BNNS, the composite film still maintains an integrated film structure, as shown in Figure 1(d1,d2). However, compared with the pure PVA film, the transparency of the BNNS/PVA composite film is significantly reduced due to the introduction of BNNS fillers, which increase light scattering within the fibrous network. The SEM image at a scale of 20 μm in Figure 1(d3) shows that the composite film retains a continuous electrospun fiber network, suggesting that 4 wt% BNNS does not severely disrupt the electrospinning process. At a higher magnification with a scale of 4 μm in Figure 1(d4), BNNS sheets can be clearly observed on the surface of nearly every fiber, resulting in a rough but continuous fibrous morphology.

These results confirm the successful incorporation of BNNS into the PVA electrospun fibers. The attached BNNS increases the surface roughness of the fibers and contributes to the formation of continuous heat-conduction pathways along the fibrous network, while the overall fiber continuity and film integrity are preserved. Therefore, the 4 wt% BNNS/PVA composite film provides a suitable structural basis for subsequent flexible thermal management applications.

## 3. Thermo-Mechanical Topology Optimization of Kirigami Structures

Flexible thermal films used in flexible electronics are required to dissipate localized heat while maintaining mechanical compliance under repeated deformation [29]. However, conventional continuous films often suffer from a trade-off between thermal transport and deformability: insufficient heat dissipation leads to local heat accumulation, whereas tensile deformation induces elastic strain energy accumulation and stress concentration [30]. To address this issue, thermo-mechanical topology optimization was introduced to regulate the in-plane material distribution of the BNNS/PVA composite film. By retaining effective heat-conduction pathways around the central heat source and removing mechanically inefficient regions, the TO kirigami structure enables simultaneous reduction of the average temperature and total elastic strain energy [31], thereby improving both heat dissipation and deformation accommodation.

### 3.1. Density Method for Thermo-Mechanical Topology Optimization

A density-based topology optimization method was employed to regulate the in-plane material distribution of the BNNS/PVA composite film. The density method follows the Solid Isotropic Material with Penalization (SIMP) framework [32], in which material properties are interpolated using a continuous elemental density variable and a penalization factor, has been widely used in structural design, heat conduction optimization, and multi-physics topology optimization. In this work, the thermo-mechanical topology optimization was performed in COMSOL Multiphysics (Version 6.2) using a density method.

For the present BNNS/PVA composite film, the design domain Ω was divided into finite elements. Each element was assigned a density variable *ρ_e_* [33], which represents the material state of the e-th element:

(2) $$0<\rho_{min}\leq \rho_{e}\leq 1$$

where *ρ_e_* = 1 indicates that the element is fully occupied by solid BNNS/PVA composite material, while *ρ_e_* = *ρ_min_* represents a void or weak material region. Here, *ρ_min_* is a very small positive value introduced to avoid numerical singularity during finite element calculation.

In the density method, the thermal and mechanical properties of each finite element are interpolated by the elemental density variable *ρ_e_*. For thermal analysis, the effective thermal conductivity of the *e*-th element is defined as follows:

(3) $$k_{e}=k_{min}+\rho_{e}^{p}(k_{solid}-k_{min})$$

where *k_e_* is the effective thermal conductivity of the element, *k_solid_* is the thermal conductivity of the solid BNNS/PVA composite, *k_min_* is a small value assigned to the void region, and *p* is the penalization factor.

Similarly, the effective elastic modulus of each element is expressed as follows:

(4) $$E_{e}=E_{min}+\rho_{e}^{p}(E_{solid}-E_{min})$$

where *E_e_* is the effective elastic modulus of the element, *E_solid_* is the elastic modulus of the solid BNNS/PVA composite, *E_min_* is a small stiffness value assigned to the void region. Therefore, the same density variable controls both the heat-conduction capability and mechanical stiffness of the composite film.

In the SIMP-based topology optimization, the penalization factor was set as *p* = 3, to encourage a discrete distribution between solid and void regions. The minimum thermal conductivity and elastic modulus were defined as *k_min_* = 10^−9^ *k_solid_* and *E_min_* = 10^−9^ *E_solid_*, respectively, to prevent numerical singularity during finite element calculations. The optimization problem was solved using the Method of Moving Asymptotes (MMA) algorithm implemented in COMSOL Multiphysics. The iterative process was terminated when the relative variation of the objective function between two consecutive iterations was less than 1%.

During each optimization iteration, finite element analysis was performed to calculate the temperature field, displacement field, stress distribution, and total elastic strain energy. The material distribution was then updated under the prescribed volume-fraction constraint:

(5) $$\frac{1}{\left|\Omega \right|}\int \rho (x)d\Omega \leq 0.5$$

where |Ω| is the area of the design domain, and *ρ*(*x*) is the density distribution in the domain. This constraint indicates that no more than 50% of the design domain was retained as solid material. As a result, material was preferentially preserved in regions contributing to heat conduction and mechanical load transfer, while inefficient regions were gradually removed to form the TO kirigami structure.

### 3.2. Design Domain and Boundary Conditions

**Figure 2:**
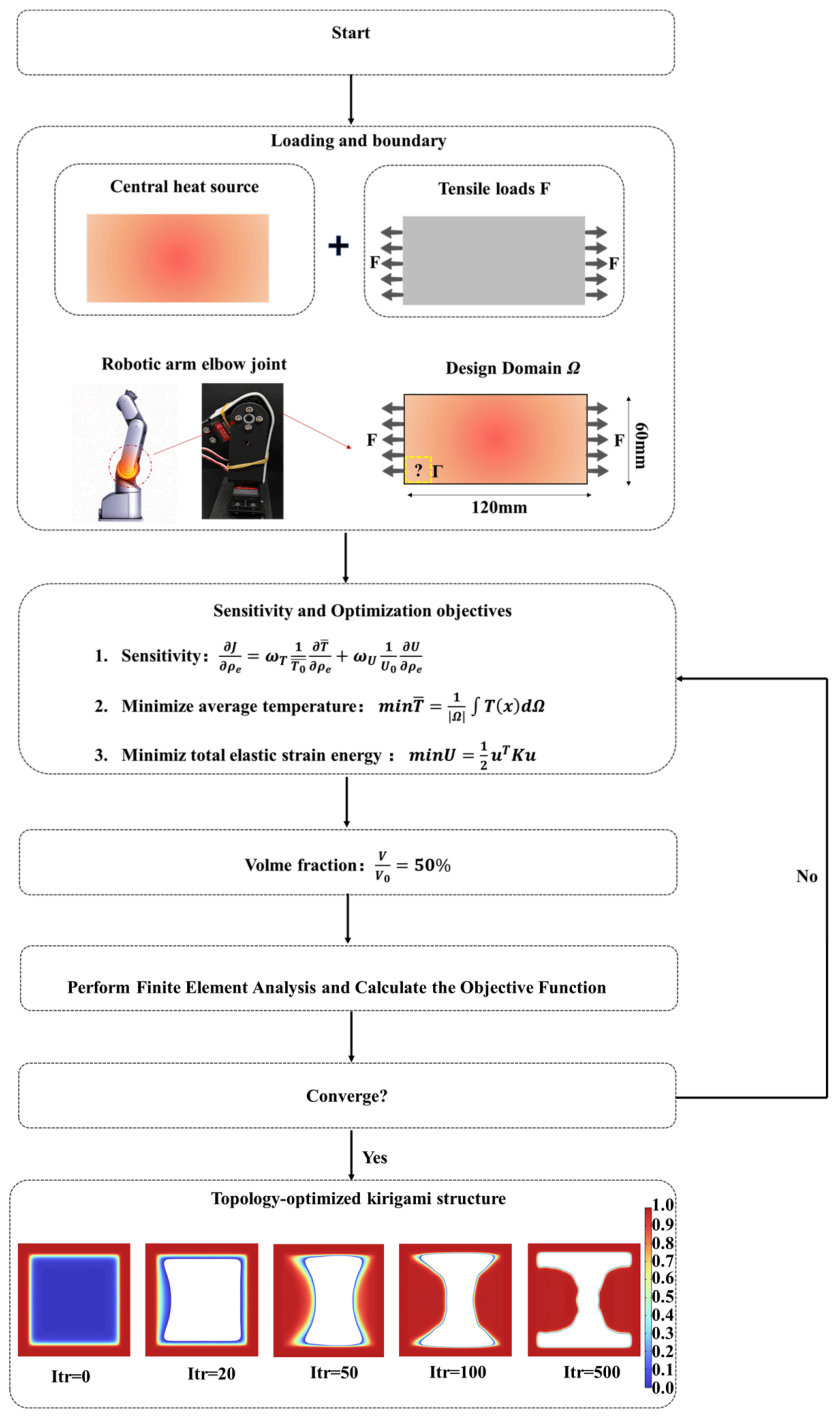
Thermo-mechanical topology optimization process of the BNNS/PVA composite film.

As shown in Figure 2, a 2D rectangular design domain Ω with a size of 120 mm × 60 mm was established to represent the planar geometry of the BNNS/PVA composite film. The mechanical behavior of the composite film was described using a linear elastic constitutive model. The Young’s modulus and Poisson’s ratio were set as 100 MPa and 0.4, respectively [34]. To reproduce the coupled thermal and mechanical loading conditions, a localized heat source was applied at the center of the design domain to simulate heat generation from local electronic components. Meanwhile, uniaxial tensile loads F were imposed on the two lateral boundaries to represent the stretching deformation experienced by the flexible film during operation.

The outer boundary of the design domain was defined as Γ, where material was preserved throughout the optimization process. This boundary constraint was introduced to maintain the edge continuity and structural integrity of the optimized film. Therefore, the optimization was mainly conducted on the internal material distribution, aiming to retain effective heat-conduction pathways from the central heat source while removing mechanically inefficient regions to improve deformation accommodation under tensile loading.

### 3.3. Optimization Objectives

The thermo-mechanical topology optimization was performed to improve both the heat dissipation capability and deformation accommodation of the BNNS/PVA composite film. In this work, two performance indicators were considered: the average temperature and the total elastic strain energy (TESE). The average temperature was used to evaluate the overall thermal response of the film, while TESE was used to characterize the elastic energy accumulation under tensile deformation.

The average temperature of the design domain is defined as follows:

(6) $$\bar{T}=\frac{1}{\left|\Omega \right|}\int T(x)d\Omega$$

where Ω is the design domain, |Ω| is the area of the design domain, and *T*(*x*) is the local temperature distribution.

The total elastic strain energy is expressed as follows:

(7) $$U=\frac{1}{2}u^{T}Ku$$

where *U* is the TESE, *u* is the global displacement vector, and *K* is the global stiffness matrix. A lower value of *U* indicates reduced elastic energy accumulation in the film under tensile deformation.

Based on these two indicators, a normalized thermo-mechanical objective function was established to simultaneously evaluate the thermal and mechanical performances:

(8) $$F=w_{T}\frac{T}{T_{0}}+w_{U}\frac{U}{U_{0}}$$

where *F* represents the normalized thermo-mechanical objective function used as the optimization criterion. *T* and *U* are the average temperature and total elastic strain energy of the optimized structure, respectively. *T*_0_ and *U*_0_ are the corresponding values of the initial structure, which are introduced for normalization to eliminate the difference in magnitude between the thermal and mechanical objectives. *w_T_* and *w_U_* denote the weighting coefficients of thermal and mechanical objectives.

In this work, the weighting coefficients were set as *w_T_* = 0.7 and *w_U_* = 0.3, respectively. The higher thermal weighting was selected because the primary objective of the proposed BNNS/PVA film is efficient thermal management, while sufficient mechanical compliance is required to maintain structural integrity during deformation.

Therefore, minimizing the combined objective function enables the optimized kirigami structure to achieve an effective balance between heat dissipation and deformation accommodation.

### 3.4. Numerical Model and Mesh Independence Analysis

The finite element model was discretized using triangular elements, and the mesh density was carefully controlled to ensure numerical accuracy. Since the thermo-mechanical topology optimization involves coupled temperature and deformation fields, the influence of mesh resolution on the simulation results was evaluated through a mesh independence analysis.

Three different mesh densities were investigated by gradually refining the element size. The average temperature and total elastic strain energy (TESE), which represent the thermal and mechanical responses of the optimized structure, were selected as evaluation indicators. As summarized in Table 1, the calculated average temperature and TESE exhibited negligible variation with further mesh refinement. When the mesh was refined from the medium-density mesh to the fine-density mesh, the relative deviations of both indicators were less than 1%, indicating that the numerical results were independent of mesh resolution.

Therefore, the medium-density mesh was adopted for subsequent thermo-mechanical topology optimization considering both computational efficiency and simulation accuracy.

### 3.5. Thermo-Mechanical Optimization Process

The thermo-mechanical topology optimization process and the corresponding material evolution are shown in Figure 2. Under a prescribed volume fraction constraint of 50%, finite element analysis was performed iteratively to evaluate the thermal and mechanical responses of the design domain. Starting from an initially continuous rectangular domain (Itr = 0), material with limited contribution to heat conduction and load transfer was progressively removed, while regions surrounding the central heat source and principal load-transfer pathways were preferentially retained. Consequently, the topology gradually evolved into a kirigami-like structure with a narrowed central region and continuous lateral pathways, enabling a balance between heat spreading and tensile deformation accommodation. The optimized unit cell converged into a dog-bone-like void configuration, which was subsequently adopted as the basic kirigami element. By periodically arranging these optimized elements according to the kirigami design principle, the final topology-optimized kirigami pattern was constructed for experimental fabrication. The structural topology became nearly stable after approximately 100 iterations, with only minor modifications observed before final convergence at Iteration 500.

To quantitatively evaluate the optimization performance, the average temperature and temperature standard deviation of the design domain were employed in this work as quantitative indicators of thermal performance and continuously tracked throughout the thermo-mechanical topology optimization process.

**Figure 3:**
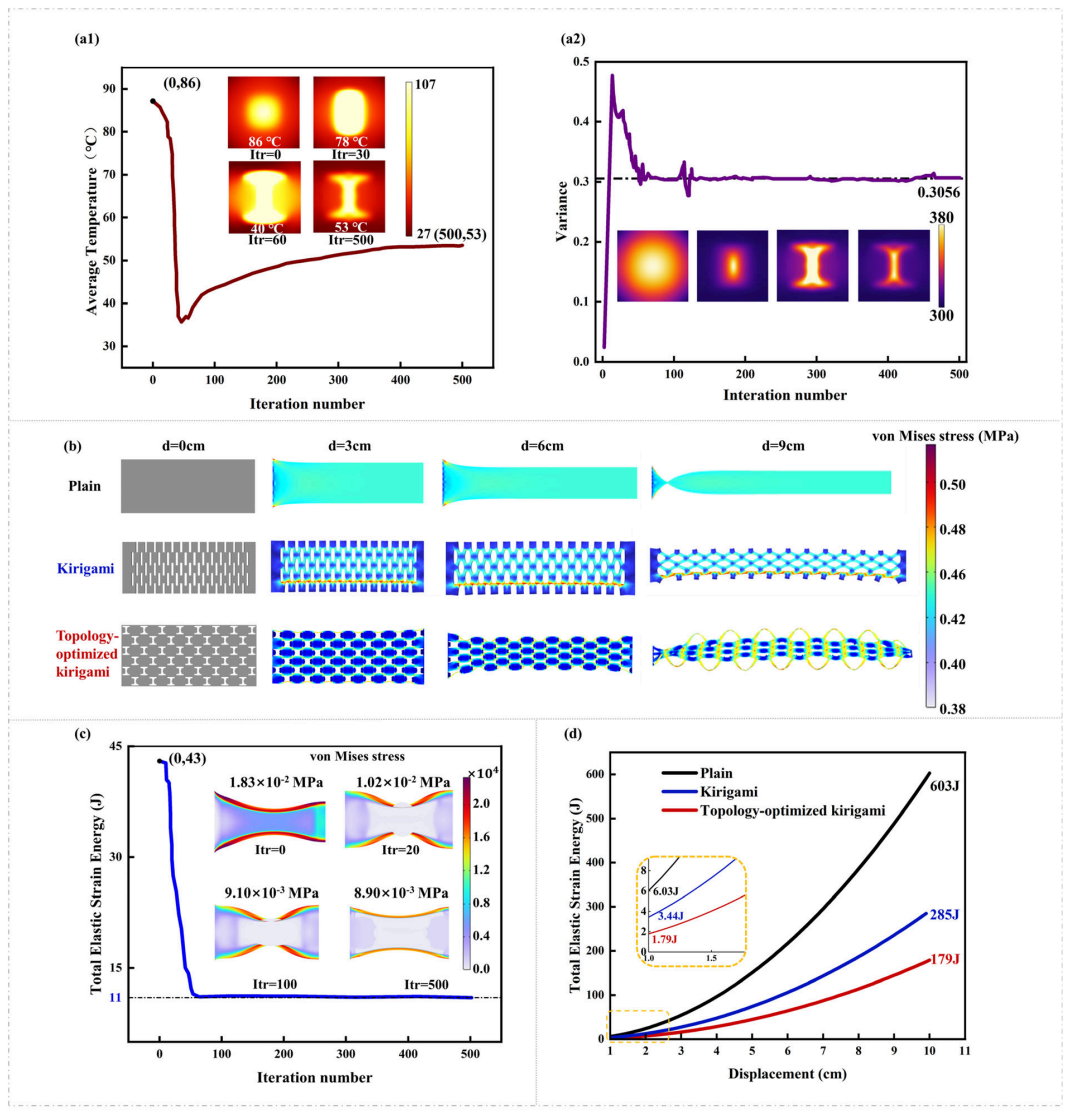
Evolution of thermal and mechanical objective functions during thermo-mechanical topology optimization and comparison of deformation responses among different structural configurations. (**a1**) Evolution of average temperature; (**a2**) Evolution of temperature standard deviation. (**b**) Deformation modes of the three structures. (**c**) Convergence of TESE. (**d**) TESE–displacement curves of the three structures.

As shown in Figure 3(a1), the average temperature of the design domain decreased rapidly during the early stage of optimization. Starting from an initial value of 86 °C at Iteration 0, the average temperature dropped to approximately 78 °C around Iteration 30 and gradually converged to 53 °C after 500 iterations. The significant reduction during the initial iterations indicates that the topology optimization effectively redistributed material around the heat source, establishing more efficient in-plane heat-conduction pathways. Although a slight increase in temperature occurred during the later optimization stage due to the simultaneous consideration of mechanical performance, the final optimized structure still exhibited a 33 °C reduction compared with the initial design.

The temperature standard deviation, defined as follows:

(9) $$\sigma_{T}={\left[\frac{1}{A}\int_{A}{\left(T(x)-T_{a}\right)}^{2}dA\right]}^{\frac{1}{2}}$$

where *T*(*x*) is the local temperature, *A* is the total area of the evaluated surface. and *T_a_* is the average temperature of the design domain, was employed to evaluate temperature uniformity. As shown in Figure 3(a2), the temperature standard deviation decreased from approximately 0.48 to 0.3056 during the optimization process and gradually converged after about 150 iterations. The continuous reduction of *σ_T_* indicates that local hot spots were progressively suppressed and the temperature field became more uniform. Therefore, the simultaneous decrease in average temperature and temperature standard deviation confirms that the thermal objective function was effectively minimized through topology optimization.

The optimized material distribution and corresponding deformation behavior are illustrated in Figure 3b. Under identical tensile displacement, the plain film exhibited severe deformation localization near the constrained boundaries, indicating poor strain accommodation capability. The conventional kirigami structure partially released the applied strain through the opening of the cut patterns, resulting in improved deformability. In contrast, the topology-optimized kirigami structure formed a highly interconnected lattice network, allowing deformation to be distributed more uniformly throughout the entire domain. As the displacement increased from 3 cm to 9 cm, the optimized structure showed larger geometric reconfiguration and reduced local stress concentration compared with the other two configurations, demonstrating superior tensile compliance.

The evolution of TESE during topology optimization is shown in Figure 3c. The initial structure exhibited a TESE of approximately 43 J. After optimization, the TESE decreased sharply during the first 50 iterations and eventually converged to approximately 11 J at Iteration 500, corresponding to a reduction of nearly 74%. The von Mises stress distributions further reveal that the maximum stress decreased from 1.83 × 10^−2^ MPa to 8.9 × 10^−3^ MPa. These results indicate that the optimized topology effectively alleviated stress concentration and reduced elastic energy accumulation under tensile loading. It should be noted that the von Mises stress shown in Figure 3c corresponds to a single optimized unit cell, while Figure 3b represents the stress distribution of the entire optimized design domain. Therefore, these two results correspond to different evaluation scales and should not be directly compared in terms of absolute stress values. The discrepancy between simulation and experiment mainly originates from the different loading conditions. The numerical model was established under idealized small-deformation conditions with uniform material properties and simplified boundary constraints, whereas the experimental tensile test involved larger deformation and practical factors such as fiber defects, interfacial interactions, and local stress concentration.

To further compare the deformation accommodation capability of different structures, the variation of TESE with tensile displacement is plotted in Figure 3d. For all structures, TESE increased monotonically with increasing displacement because larger deformation induced greater elastic energy storage. However, substantial differences were observed among the three configurations. At a displacement of 10 cm, the plain film exhibited the highest TESE of approximately 603 J, whereas the kirigami structure reduced the value to 285 J. The topology-optimized kirigami structure achieved the lowest TESE of only 179 J. Compared with the plain film and conventional kirigami structure, the optimized design reduced TESE by approximately 70% and 37%, respectively. The enlarged view at small displacements further demonstrates that the optimized structure consistently maintained the lowest elastic energy accumulation throughout the entire deformation process. This behavior confirms that the topology-optimized kirigami architecture can effectively redistribute deformation, reduce stress concentration, and improve mechanical compliance while preserving efficient thermal transport pathways.

## 4. Experiments

Robotic joints and other deformable electronic systems often suffer from heat accumulation under continuous operation while simultaneously undergoing large mechanical deformation [35]. Although two-dimensional thermally conductive fillers can improve the thermal transport capability of flexible films [36], maintaining thermal management performance under repeated bending and stretching remains challenging. By combining BNNS-based composite films with TO kirigami structures, both thermal dissipation and mechanical compliance can be achieved [37].

### 4.1. Thermal Dissipation Performance of BNNS/PVA Composite Films for Flexible Electronics

**Figure 4:**
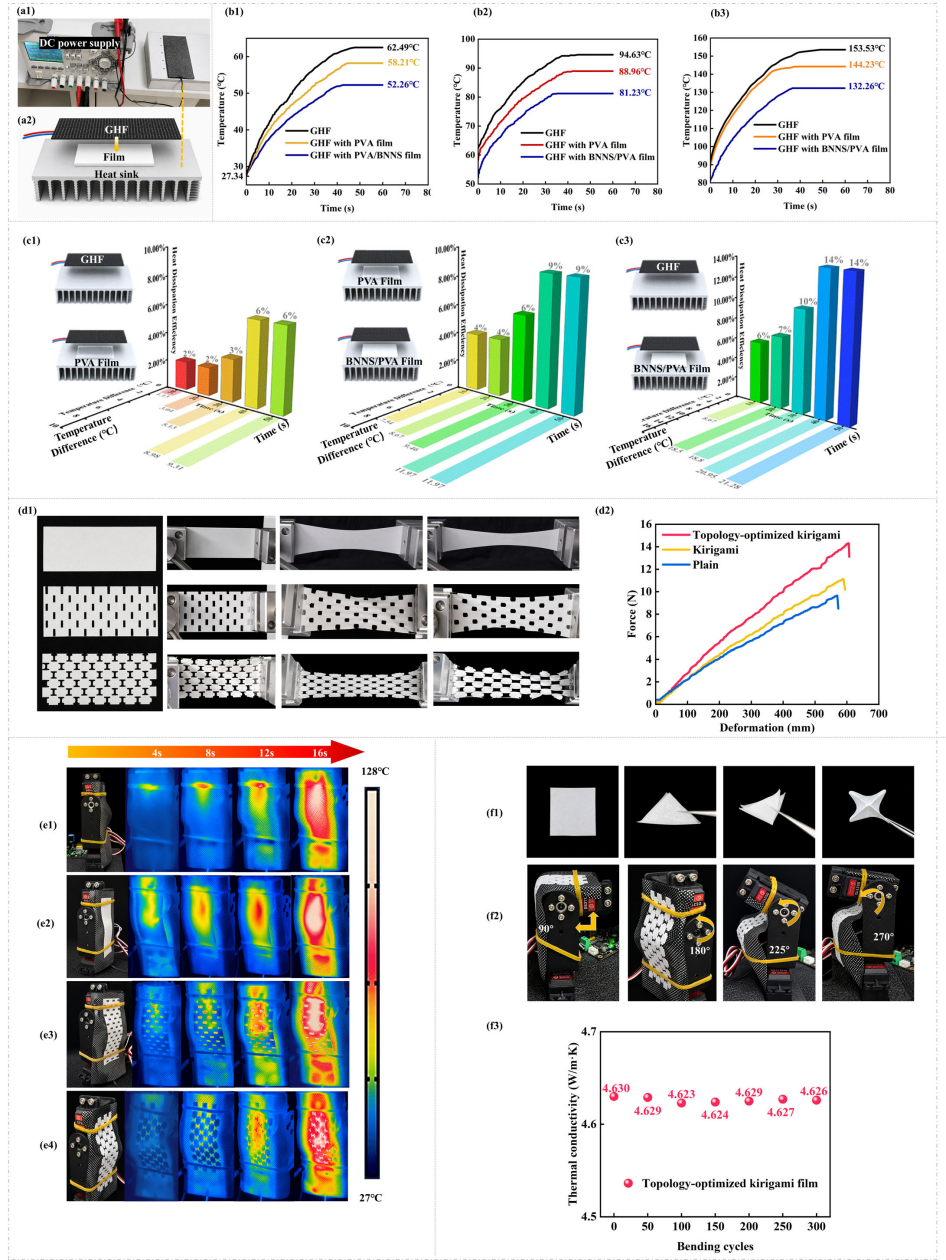
Thermo-mechanical performance and robotic-joint thermal management of kirigami-engineered BNNS/PVA composite films. (**a**) Thermal dissipation system. (**b**) Temperature rise curves at (**b1**) 1.5 W, (**b2**) 2.4 W and (**b3**) 3.3 W. (**c**) Temperature differences and heat dissipation efficiencies at 3.3 W: (**c1**) GHF vs. GHF + PVA, (**c2**) PVA vs. BNNS/PVA, (**c3**) GHF vs. GHF + BNNS/PVA. (**d1**) Tensile deformation behavior three structures. (**d2**) Force-Deformation curves of the three structures. (**e1**–**e4**) Thermal management performance on a robotic elbow joint. (**f1**) The flexibility of the BNNS/PVA film. (**f2**) TO kirigami BNNS/PVA film bent on a robotic elbow joint. (**f3**) In-plane thermal conductivity of the TO kirigami BNNS/PVA film in response to 300 bending cycles.

The thermal management performance of the electrospun BNNS/PVA composite films was evaluated using a heating platform, as illustrated in Figure 4(a1,a2). Figure 4(a1) shows the experimental platform, whereas Figure 4(a2) schematically illustrates the vertical assembly, in which the GHF was placed above the composite film and the composite film was attached to the aluminum heat sink to establish the heat-transfer pathway. PVA and BNNS/PVA films were cut into uniform 60 mm × 60 mm squares and mounted onto a heat sink. Above each film, a Graphene Heating Film (GHF) was placed as a flexible heat source driven by a DC power supply to deliver constant input power. The tested film was positioned between the GHF and an aluminum heat sink, allowing heat to be dissipated through the thermal-management layer. This configuration was designed to simulate the heat-transfer process commonly encountered in flexible electronic devices.

Quantitative temperature evolution under multiple constant power inputs is summarized in Figure 4b. At an input power of 1.5 W (Figure 4(b1)), starting from an ambient temperature of 27.34 °C, the steady-state temperatures for GHF, GHF with PVA film, and GHF with BNNS/PVA film were 62.49 °C, 58.21 °C, and 52.26 °C, respectively. The BNNS/PVA film achieved a 10.23 °C drop relative to the bare GHF. When tested at 2.4 W (Figure 4(b2)), the corresponding steady-state temperatures were approximately 94.63 °C, 88.96 °C, and 81.23 °C, yielding a 13.4 °C reduction with BNNS/PVA. At 3.3 W (Figure 4(b3)), these values further increased to roughly 153.53 °C, 144.23 °C, and 132.26 °C, with a 21.27 °C reduction.

In Figure 4c, temperature differences under 3.3 W were analyzed to estimate heat dissipation efficiency *η*:

(10) $$\eta =\frac{hA\Delta T}{P_{in}}\times 100\%$$

where *h* = 5.9 W/(m^2^·K) is the convective heat transfer coefficient [38], *A* is the sample area (60 mm × 60 mm), Δ*T* is the temperature difference between, and *P_in_* is the input power.

Compared with the bare GHF, the PVA film produced a temperature reduction of 9.31 °C, corresponding to a heat dissipation efficiency of approximately 6% (Figure 4(c1)). Upon incorporating BNNS into the PVA matrix, the temperature difference further increased to 11.97 °C, resulting in an additional efficiency improvement of about 8% (Figure 4(c2)). As a result, the BNNS/PVA composite film achieved a total temperature reduction of 21.27 °C relative to the uncovered GHF, corresponding to an overall heat dissipation efficiency of approximately 14% (Figure 4(c3)). These results indicate that the synergistic effect of the electrospun fibrous network and the highly thermally conductive BNNS facilitates the formation of continuous heat-transfer pathways, leading to more efficient heat spreading and enhanced thermal-management capability.

### 4.2. Tensile Deformation Behavior of Plain, Kirigami, and TO Kirigami Structures

Figure 4(d1) presents the tensile deformation processes of the plain film, conventional kirigami film, and TO kirigami film. The plain film exhibited limited deformability and rapidly developed localized strain concentration during stretching, resulting in restricted elongation. After introducing the kirigami pattern, the deformation mode changed from material stretching to structural deformation, allowing the cut units to rotate, open, and rearrange progressively under tensile loading. Consequently, the kirigami film demonstrated a larger deformation capability than the plain film. In contrast, the TO kirigami film showed the most uniform deformation behavior throughout the stretching process. Owing to the optimized distribution and geometry of the kirigami units, the applied strain was more effectively dispersed over the entire structure, reducing local stress concentration and enabling greater structural compliance. The deformation images clearly demonstrate that the TO architecture can accommodate larger tensile deformation while maintaining structural integrity.

The corresponding force–deformation curves are shown in Figure 4(d2). All three samples exhibited a nearly linear increase in tensile force with deformation before reaching their peak loads. The TO kirigami structure achieved the highest maximum load (*F*_max_) of 14.32 N, compared with 11.13 N for the kirigami structure and 9.64 N for the plain structure. To further evaluate the mechanical performance, the maximum engineering stress (*σ_max_*) was calculated according to:

(11) $$\sigma_{max}=\frac{F_{max}}{A}$$

where *A* is the cross-sectional area of the specimen. Based on the specimen dimensions (120 mm × 60 mm × 0.1 mm), the cross-sectional area was determined to be 6 mm^2^. Accordingly, the maximum stresses of the TO kirigami, kirigami, and plain structures were calculated to be 2.39 MPa, 1.86 MPa, and 1.61 MPa, respectively.

The maximum engineering strain (*ε_max_*) was calculated using:

(12) $$\varepsilon_{max}=\frac{\Delta L}{L_{0}}\times 100\%$$

where Δ*L* is the maximum deformation at the peak load and *L*_0_ is the initial gauge length of the specimen. Based on an initial length of 120 mm, the maximum deformations of the TO kirigami, kirigami, and plain structures were approximately 602 mm, 581mm, and 562 mm, respectively. Accordingly, the corresponding maximum strains were calculated to be 502%, 484%, and 468%.

The tensile properties of the three structures are summarized in Table 2; the TO kirigami structure achieved the highest maximum stress (2.39 MPa) and maximum strain (5.02%) among all samples. Compared with the plain structure, the maximum stress increased by 48.4%, while the maximum strain was simultaneously improved from 4.68% to 5.02%. These results indicate that topology optimization not only enhances the load-bearing capability of the structure but also preserves its stretchability. The ability to achieve higher strength without compromising deformation capacity highlights the effectiveness of the optimized kirigami architecture for flexible electronic thermal-management applications.

### 4.3. Thermal Management Performance and Mechanical Reliability Under Robotic Joint Deformation

To further evaluate the practical applicability of the developed films in deformable thermal-management systems, robotic elbow-joint experiments were conducted. As shown in Figure 4e, four different configurations, including bare GHF, plain film-covered GHF, kirigami film-covered GHF, and TO kirigami film-covered GHF, were mounted on a robotic joint and subjected to identical heating conditions.

The surface temperature distribution was captured using a Forward-Looking Infrared (FLIR). For the bare GHF (Figure 4(e1)), heat was highly concentrated near the heating region, resulting in pronounced local hot spots and severe temperature accumulation during operation. After introducing the plain BNNS/PVA film (Figure 4(e2)), the average temperature was reduced and heat spreading was moderately improved. The kirigami film (Figure 4(e3)) further enhanced heat distribution owing to its enlarged deformation space and improved surface conformability. Notably, the TO kirigami film (Figure 4(e4)) exhibited the most uniform temperature field throughout the heating process. The optimized kirigami architecture enabled more effective heat spreading across the entire film surface, thereby suppressing local thermal concentration and improving thermal-management efficiency under dynamic deformation conditions.

A quantitative comparison between the experimental and simulated thermal responses is presented in Table 3. Under identical heating conditions, the experimentally measured average surface temperature decreased from 95 °C for the bare GHF to 68 °C after covering it with the TO kirigami film, corresponding to a temperature reduction of 27 °C. The numerical simulation predicted a decrease from 86 °C to 53 °C, corresponding to a reduction of 33 °C. Although the simulated temperatures were lower than the experimentally measured values, both results exhibited the same trend and confirmed that the TO kirigami structure provided the most effective suppression of heat accumulation. The difference between the experimental and simulated temperature reductions was only 6 °C. The remaining discrepancy in absolute temperature may be attributed to idealized thermal boundary conditions, uncertainties in the convective heat-transfer coefficient and material properties, interfacial thermal contact resistance, surface-emissivity variations, and unavoidable fabrication deviations. Overall, the consistent temperature-reduction trend observed in the experiment and simulation supports the reliability of the numerical model for predicting the thermal management performance of the optimized kirigami structure.

To distinguish the contributions of material enhancement and structural optimization, the thermal management performance of different configurations was quantitatively compared, as summarized in Table 4. Compared with the pure PVA film, the incorporation of BNNS resulted in the largest temperature reduction of 12 °C, demonstrating that the enhanced in-plane thermal conductivity of BNNS plays a dominant role in improving heat dissipation. Furthermore, the kirigami structure and topology optimization provided additional temperature reductions of 7 °C and 8 °C, respectively, by improving heat spreading and deformation adaptability. These results demonstrate the synergy between BNNS enhancement and kirigami structural optimization.

The flexibility of the BNNS/PVA composite film is demonstrated in Figure 4(f1). The film can be folded, twisted, and deformed into various shapes without visible structural damage, indicating excellent mechanical compliance. To assess its suitability for robotic applications, the TO kirigami composite film was attached to the elbow joint of a robotic arm and subjected to continuous bending from 90° to 270°, as shown in Figure 4(f2). Throughout the entire bending process, the film remained firmly attached to the joint surface without delamination or fracture, demonstrating its ability to accommodate large-angle deformation while maintaining structural integrity.

The durability of the TO kirigami composite film was further evaluated through cyclic bending tests. The bending deformation was generated using a DS3218MG digital servo, which was programmed to repeatedly bend the sample for 300 cycles under identical deformation conditions. After the cyclic bending test, the in-plane thermal conductivity was measured using the same procedure described above to evaluate the thermal stability of the composite film. As shown in Figure 4(f3), the in-plane thermal conductivity remained nearly constant after 300 bending cycles, varying only from 4.630 W/(m·K) to 4.624 W/(m·K), corresponding to a fluctuation of less than 0.2%. The negligible change in in-plane thermal conductivity indicates excellent thermo-mechanical stability and repeatability under repeated deformation. These results collectively demonstrate that the topology-optimized kirigami composite film combines efficient heat dissipation, large deformation capability, and good short-term cyclic stability, making it a promising candidate for thermal-management applications in flexible electronics and robotic systems.

## 5. Conclusions

With the aim of addressing the challenge of achieving efficient heat dissipation and mechanical compliance in flexible electronics, a TO kirigami BNNS/PVA composite film was developed by integrating BNNS incorporation, electrospinning, and thermo-mechanical topology optimization. The electrospun BNNS/PVA network increased the in-plane thermal conductivity from 0.19 to 4.63 W/(m·K), representing an enhancement of 2337%. The optimized kirigami architecture reduced the average temperature from 359 K to 326 K and decreased the total elastic strain energy from 43 J to 11 J. Under identical heating conditions, the composite film achieved a maximum temperature reduction of 21.27 °C with a heat-dissipation efficiency of approximately 14%.

In addition, the TO kirigami structure exhibited a maximum stress of 2.39 MPa and a maximum strain of 5.02%, while maintaining stable in-plane thermal conductivity after 300 bending cycles. These results demonstrate that the combination of thermally conductive BNNS/PVA films and topology-optimized kirigami structures provides an effective strategy for thermal management in flexible electronics and soft robotic systems.

## Figures and Tables

**Table 1:** Mesh independence analysis of the thermo-mechanical simulation.

Mesh Size	Maximum Element Size (mm)	T_average_ (°C)	TESE (J)
Extra Coarse	5	53.2	11.31
Medium	2.5	53.0	11.25
Extra Fine	1.25	52.9	11.09

**Table 2:** Comparison of Tensile Mechanical Properties of Three Structures.

Structure	*F_max_* (N)	*σ_max_* (MPa)	Δ*L* (mm)	*ε_max_* (%)
Plain	9.64 ± 0.32	1.61 ± 0.06	562 ± 12	468 ± 10
Kirigami	11.13 ± 0.38	1.86 ± 0.09	581 ± 15	484 ± 12
TO Kirigami	14.32 ± 0.41	2.39 ± 0.11	602 ± 16	502 ± 13

The initial gauge length *L*_0_ = 120 mm. The nominal stress was calculated using the original gross cross-sectional area.

**Table 3:** Comparison of Surface Temperature Reduction for Different Structures.

Structure	Experimental *T_average_* (°C)	Simulated *T_average_* (°C)	Experimental Δ*T* (°C)	Simulated Δ*T* (°C)
GHF	95 ± 1.5	86	-	-
Plain	81 ± 1.2	-	14	-
Kirigami	74 ± 0.8	-	21	-
TO Kirigami	68 ± 0.3	53	27	33

Δ*T* represents the reduction in average surface temperature relative to the bare GHF.

**Table 4:** Contribution analysis of material and structural optimization on thermal management performance.

Configuration	Δ*T* (°C)
GHF with BNNS/PVA film	12
GHF with Kirigami BNNS/PVA film	7
GHF with TO Kirigami BNNS/PVA film	8

## Data Availability

The original contributions presented in this study are included in the article. Further inquiries can be directed to the corresponding authors.
